# A Case of Breast Cancer Recurrence Mimicking a Thymus Tumor With Vascular Invasion

**DOI:** 10.7759/cureus.91311

**Published:** 2025-08-30

**Authors:** Takaya Sato, Kentaro Minegishi, Naota Okabe, Masaya Sogabe, Hiroyoshi Tsubochi, Shunsuke Endo

**Affiliations:** 1 Department of General Thoracic Surgery, Jichi Medical University Saitama Medical Center, Saitama, JPN; 2 Department of Pathology, Jichi Medical University Saitama Medical Center, Saitama, JPN

**Keywords:** adult thoracic surgery, anterior mediastinal tumor, late recurrence of breast cancer, lymphatic drainage, superior vena cava tumor thrombosis

## Abstract

Mediastinal tumors have many differential diagnoses, making it challenging to confirm a diagnosis through imaging alone. We report a case of mediastinal recurrence of breast cancer mimicking a thymic tumor, with invasion of the left innominate vein, occurring 13 years post surgery. Given the tumor thrombus extending into the superior vena cava (SVC), surgical resection was performed to prevent life-threatening complications and to achieve definitive diagnosis and treatment. Breast cancer is known for its slow growth and late recurrence. While axillary lymph nodes (ALNs) are the most common site of breast cancer drainage, alternative pathways such as the internal mammary lymph nodes may also play a role in disease spread. Recurrence of breast cancer in IMLN can mimic mediastinal tumors, such as thymoma or thymic cancer. Therefore, in patients with a history of breast cancer, a mediastinal tumor should raise suspicion for breast cancer recurrence, even if it involves the innominate vein or occurs after a long interval.

## Introduction

Mediastinal tumors represent a diagnostic challenge due to their wide range of differential diagnoses, including thymic epithelial tumors, lymphomas, germ cell tumors, and metastatic malignancies [1]. Breast cancer is one of the most common malignancies in women worldwide and is known for its late recurrence, sometimes decades after the initial diagnosis. Although breast cancer typically spreads through the axillary lymphatic system, a small portion, approximately 3%, drains via the internal mammary lymphatic nodes (IMLN), which are located along the internal thoracic vessels. These nodes eventually drain into the innominate venous system. Metastasis via this route can result in mediastinal recurrence and even direct invasion of major vessels such as the innominate vein, potentially mimicking primary mediastinal tumors. Hormone receptor-positive breast cancer, in particular, is known to exhibit a prolonged clinical course, with potential for recurrence more than 10 or even 20 years after initial treatment [2]. Although the proportion of patients with IMLN metastases is small, these cases are occasionally encountered in clinical practice, reflecting the overall high incidence of breast cancer. We encountered a case of mediastinal recurrence of breast cancer mimicking a thymic tumor, with invasion of the left innominate vein. Recurrence of breast cancer in the IMLNs can mimic primary mediastinal tumors such as thymoma or thymic carcinoma. This report presents radiologic images and surgical findings that provide insight into the invasion route of breast cancer.

## Case presentation

**Figure 1:**
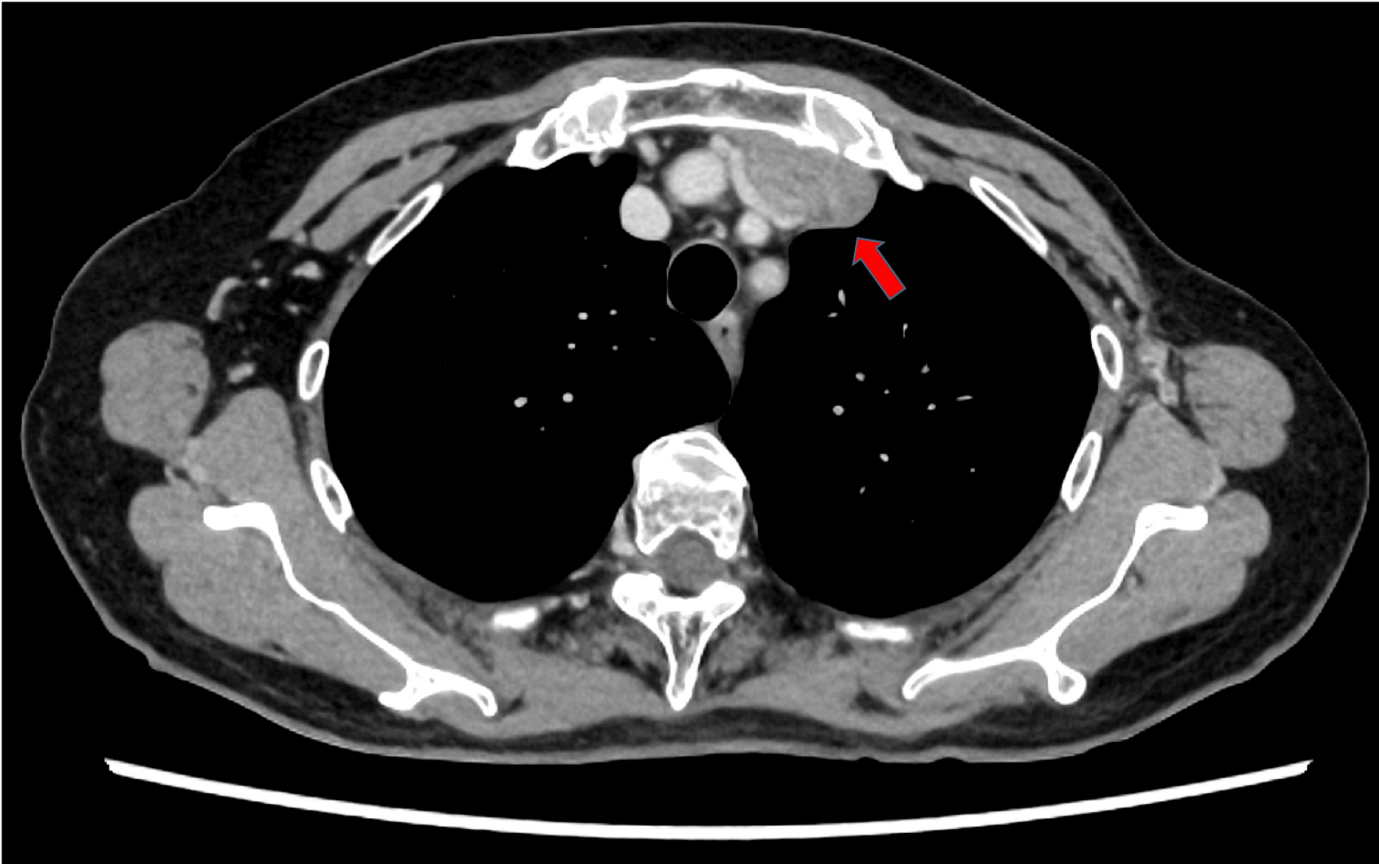
Mediastinal mass with vascular invasion on preoperative CT A mediastinal tumor (44x40x37mm) with invading the left innominate vein (red arrow).

**Figure 2:**
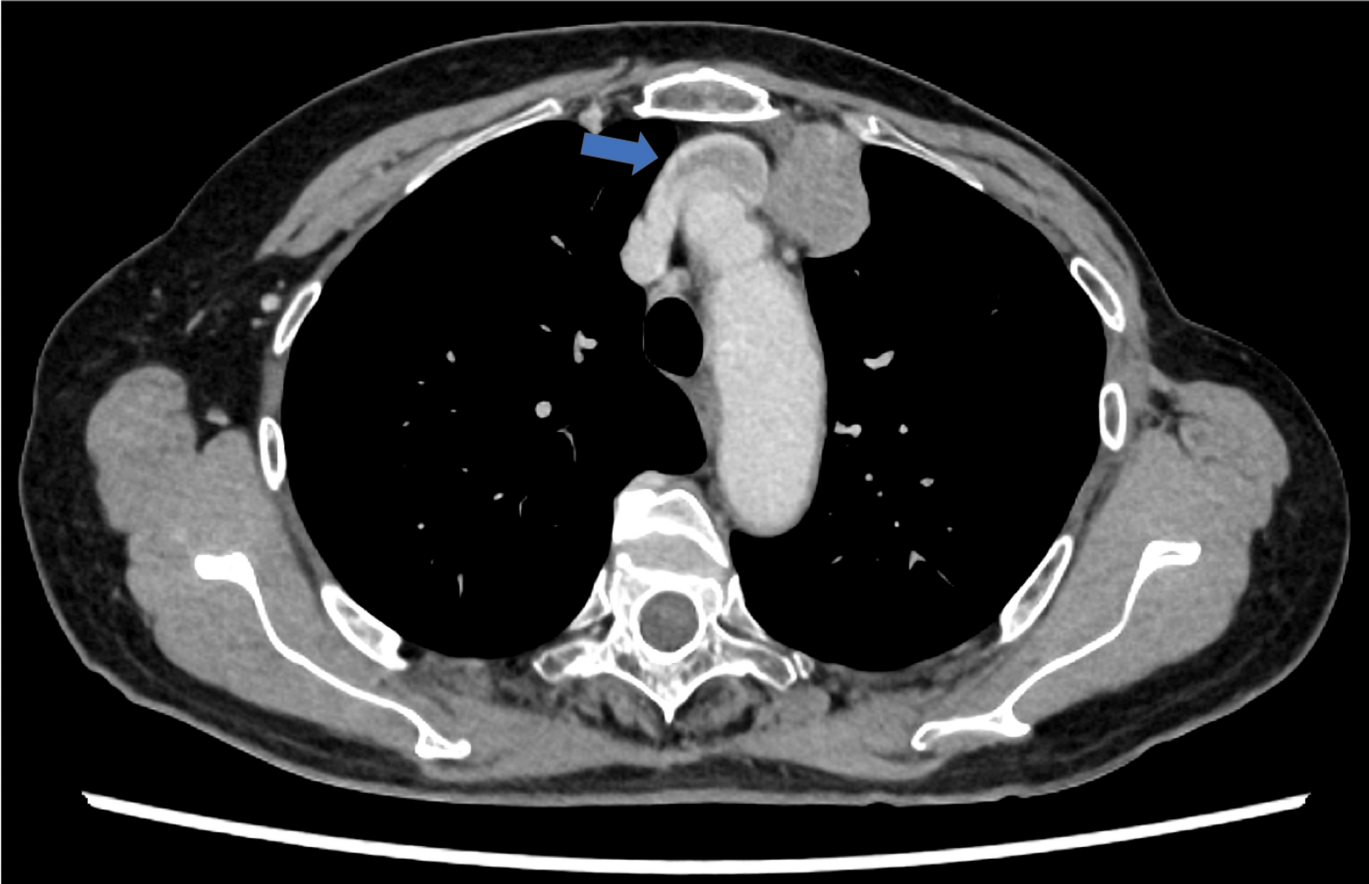
Tumor thrombus extending into the SVC on axial CT A tumor thrombus extending from the left brachiocephalic vein to the SVC (blue arrow). The PET-CT showed FDG accumulation in the tumor thrombus. SVC: superior vena cava; FDG: fluorodeoxyglucose

The patient was a 72-year-old woman who presented to our hospital with an incidentally discovered anterior mediastinal tumor. Her medical history included rectal cancer (pT3N2M0 Stage IIIa, surgery at age 57), thyroid cancer (stage unknown, surgery at age 58), and left breast cancer (pT1N0M0 Stage I, surgery at age 59). Computed tomography (CT) and 2-deoxy-2-[18F] fluoro-D-glucose positron emission tomography CT revealed a 44x40x37 mm solid tumor (maximum standardized uptake value (SUVmax) of 26.629) invading the left innominate vein, with tumor thrombus extending to the superior vena cava (SVC) (Figures 1, 2). No significant abnormalities were found in blood tests, including tumor markers (Table 1).　

**Table 1:** Preoperative laboratory test results WBC: white blood cell count; Hb: hemoglobin;  PLT: platelet count; TP: total protein; AST: aspartate aminotransferase; ALT: alanine aminotransferase; LDH: lactate dehydrogenase; CPK: creatine phosphokinase; ALP: alkaline phosphatase (IFCC method); γ-GTP: gamma-glutamyl transpeptidase; CRP: C-reactive protein; Na: sodium; K: potassium; Cl: chloride; Ca: calcium; P: phosphorus; BUN: blood urea nitrogen; TSH: thyroid-stimulating hormone; Free T3: free triiodothyronine; Free T4: free thyroxine; AChR antibody: anti-acetylcholine receptor antibody; Soluble IL-2R: soluble interleukin-2 receptor; AFP: alpha-fetoprotein; CEA: carcinoembryonic antigen; CYFRA: cytokeratin 19 fragment; NSE: neuron-specific enolase; SCC: squamous cell carcinoma antigen

Test	Tesult	Unit	Reference value
WBC	6860	/μl	3500-9100
Hb	14.1	g/dl	11.3-15.2
PLT	272000	/μl	130000-369000
TP	8.2	g/dl	6.6-8.1
Albumin	4.4	g/dl	4.1-5.1
Total bilirubin	0.54	mg/dl	0.4-1.5
Direct bilirubin	0.19	mg/dl	0.05-0.23
AST	15	U/L	13-30
ALT	13	U/L	7-20
LDH	183	U/L	124-222
CPK	47	U/L	41-153
ALP-IFCC	85	U/L	28-113
γ-GTP	13	U/L	9-32
CRP	0.02	mg/dl	0-0.14
Na	143	mmol/dL	138-145
K	4.5	mmol/dL	3.6-4.8
Cl	106	mmol/dL	100-110
Ca	9.9	mg/dl	8.4-10.1
P	3	mg/dl	2.7-4.6
BUN	12	mg/dl	8-20
Creatinin	0.61	mg/dl	0.46-0.79
TSH	0.241	μIU/ml	0.61-4.23
Free T4	1.81	ng/dl	0.71-1.69
Free T3	3	pg/dl	1.72-3.44
Anti-AChR antibodies	<0.2	nmol/l	0-0.2
Soluble IL-2R antibodies	246	U/ml	157-474
AFP	2.3	ng/ml	0-20
CEA	5.6	ng/ml	0-5
CYFRA	2.4	ng/ml	0-3.5
NSE	11.9	ng/ml	0-10
SCC	0.6	ng/ml	0-2.0

Percutaneous biopsy was not possible due to vascular invasion and the associated risk of bleeding. Although there were various differential diagnoses, we decided to perform surgery considering the risk of sudden death due to tumor thrombus in the SVC.

**Figure 3:**
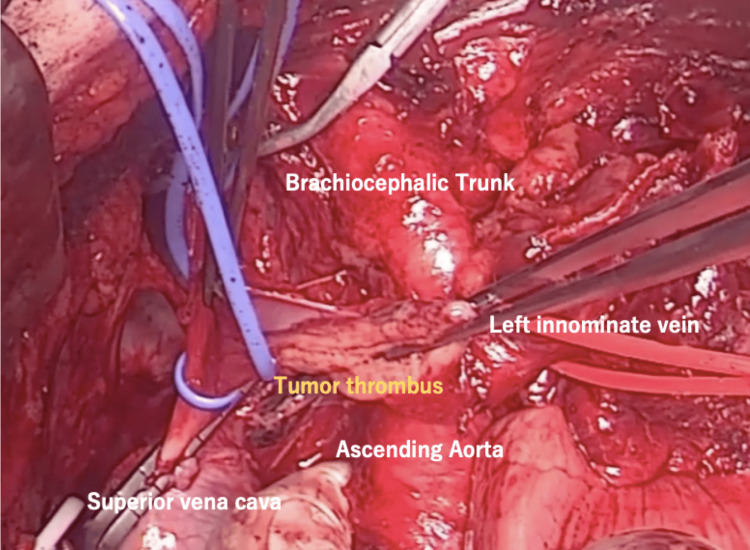
Operative findings Operative findings show the extension of the tumor thrombus into the SVC. The left innominate vein was incised after clamping, and the tumor thrombus was extracted. SVC: superior vena cava

Surgery was performed through a combined median sternotomy and the left second intercostal transverse incision. A mediastinal tumor had invaded the upper lobe of the left lung, the lateral surface of the sternum and the first costal cartilage, and the left innominate vein, so a combined resection was performed. Especially in areas where bone invasion was suspected, the periosteum was excised in combination with the periosteum. The left and right innominate veins and the SVC were temporarily clamped, and then the tumor thrombus was removed (Figure 3).

**Figure 4:**
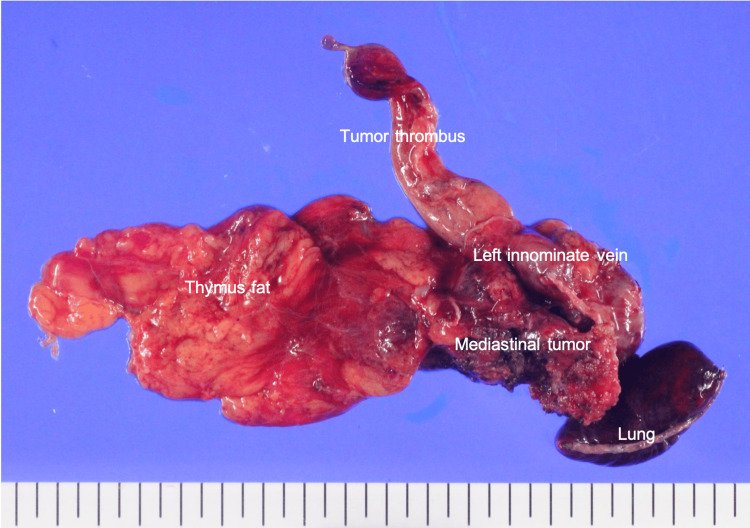
Gross specimen: mediastinal tumor with tumor thrombus The resected specimen includes the mediastinal tumor with tumor thrombus, showing direct invasion into the thymic fat, the left innominate vein, and the left upper lobe of the lung, which were resected en bloc.

**Figure 5:**
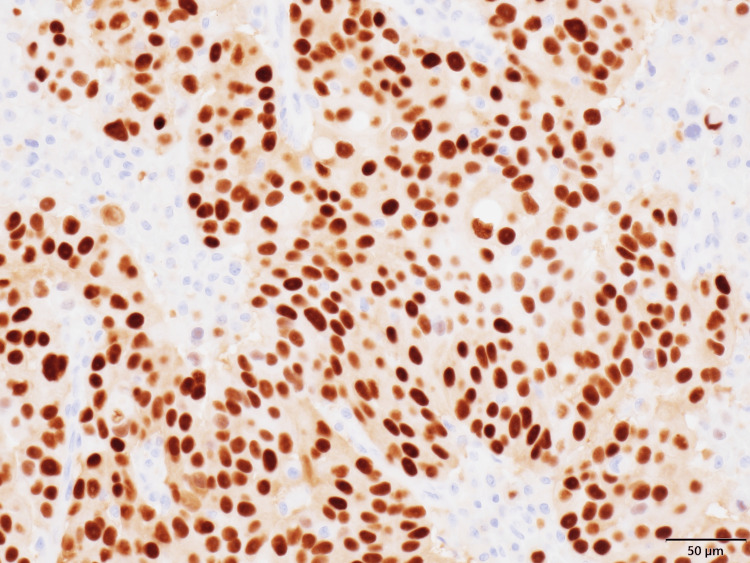
Immunohistochemical staining showing estrogen receptor tumor cells (200×) Progesterone receptor staining shows positive tumor cells (positivity rate is >90%, Allred score: 8).

**Figure 6:**
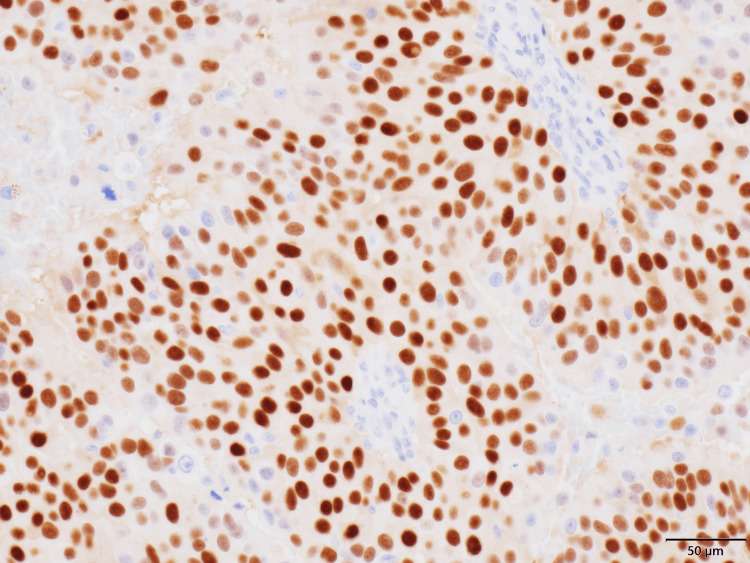
Immunohistochemical staining showing progesterone receptor (PgR)-positive tumor cells (200×) PgR staining shows positive tumor cells(positivity rate is >90%, Allred score: 8).

The operative time was 212 minutes. Blood loss was 410 ml. Gross examination revealed a mediastinal tumor infiltrating the left upper lobe and left innominate vein (Figure 4). Histopathological examination revealed an epithelial tumor consistent with adenocarcinoma. Malignant lymphoma was excluded, and the findings were not typical of germ cell tumors. The histology was also atypical for thymic epithelial tumors. The various hormone receptor profiles showed the tumor was negative for CD5, CK5/6, c-kit, and p63, while positive for estrogen receptor (ER), progesterone receptor (PgR), and GATA-3. GCDFP-15 was focally positive (Figures 5, 6). Based on these findings, she was diagnosed with recurrent breast cancer.

The postoperative course was favorable, with drain removal on postoperative day 12 and discharge on postoperative day 20. Since discharge, she has been taking letrozole 2.5 mg orally once daily as first-line therapy for this postmenopausal patient with hormone receptor-positive metastatic breast cancer. No recurrence has been observed at 16 months of follow-up. 

## Discussion

There are different kinds of metastases in breast cancer: local, regional, and distant metastases. Local metastases occur in tissues adjacent to the primary tumor, whereas regional metastases involve nearby lymph nodes. Distant metastases develop in organs located far from the original site. Common sites of breast cancer spread include lymph nodes, bones, lungs, liver, skin, and brain [3]. This case is classified as regional metastasis with adjacent organ invasion.

Lymphatic drainage from the breast is mainly directed to the axillary lymph nodes (ALNs) and, to a lesser extent, the IMLNs. The clinical significance of IMLN metastasis and its optimal management remain controversial [4]. Approximately 97% of lymphatic drainage is to the ALNs, while only about 3% flows to the IMLNs [5]. The internal mammary lymphatics run alongside the internal thoracic artery (ITA) and internal thoracic veins (ITVs) near the sternal border within the anterior intercostal spaces [6]. These nodes are most commonly found lateral to the second intercostal artery. This anatomical pathway may explain unusual patterns of direct tumor invasion.

In this case, the ITV drains directly into the left innominate vein, providing a plausible pathway for tumor spread. Although recurrence in the thymic lymph node with subsequent invasion via the thymic vein was also possible, surgical findings indicated more extensive tumor infiltration of the chest wall than the mediastinal tissues. Therefore, direct invasion along the ITV route appears more likely. Some studies suggest that aggressive resection of pulmonary and mediastinal involvement can improve local control and overall survival in select patients [7]. Therefore, awareness of this anatomical relationship is critical for surgical planning and intraoperative decision-making.

Pathologically, several differential diagnoses were considered. Primary thymic epithelial tumors were ruled out as the histology was not typical and the tumor was immunohistochemically negative for c-kit and CD5, both of which are commonly expressed in thymic carcinoma [8]. Malignant lymphoma was ruled out by the absence of lymphoid cell proliferation. Germ cell tumors were also excluded because the tumor lacked their characteristic histological patterns. In combination with the pathological findings and the patient’s history of breast cancer, the final diagnosis of metastatic breast carcinoma was made.

It is well known that even early-stage breast cancer can recur decades after initial treatment. A previous study reported a cumulative recurrence rate of 12.7% at 10 to 25 years for patients with T1N0 disease [9]. As illustrated by this case, the presence of a mediastinal mass involving the innominate vein in patients with a history of breast cancer should raise suspicion for late recurrence, regardless of the long interval since initial treatment. This underscores the importance of long-term vigilance in follow-up, especially for hormone receptor-positive tumors. Clinicians should consider prior breast cancer history even when the recurrence occurs in anatomically unexpected regions, such as the mediastinum.

## Conclusions

This case demonstrates that late recurrence of breast cancer can involve unusual lymphatic pathways, including direct invasion into the innominate vein via the ITV. Recognition of such atypical spread is important when mediastinal tumors are encountered in patients with a history of breast cancer, even long after initial treatment. Careful pathological evaluation was essential to exclude other primary mediastinal tumors and confirm metastatic breast carcinoma. For thoracic surgeons and oncologists, this case underscores the need for long-term vigilance, consideration of prior cancer history, and awareness of rare anatomical routes of recurrence to ensure accurate diagnosis, appropriate surgical planning, and optimal patient care.
